# Public Health Report

**Published:** 1919-03-15

**Authors:** 


### PUBLIC HEALTH REPORT.

### Bournemouth's Tuberculosis Scheme.

IN a short and concise report, Dr. Edwards, medical officer of health of Bournemouth, gives an account of the health of the borough during 1917. The estimated civil population was 70,327, compared with 78,395 for the previous year. The birth-rate shows a considerable fall, being 12.49, and the infant mortality has risen from 69.63 to 83.72. There is a slight fall in the death-rate, which is 15.01, compared with 15.56 for 1916. The zymotic death-rate is 0.38 per 1,000, and the death-rate from pulmonary tuberculosis 1.20 per 1,000. During the year there have been increased developments with regard to the scheme for the prevention and treatment of tuberculosis. The wise course has been adopted of detecting and treating "suspects" amongst children by the opening of the dispensary on an additional afternoon every week, and, by the provision of special sanatorium accommodation for such cases. In addition, the accommodation for the isolation of advanced cases has been increased, while a tuberculosis after-care committee has been formed, and the provision of a farm colony is contemplated. Dr. Edwards places his finger on the weak link in many tuberculosis schemes when he says, "the advanced cases demand greater attention and more adequate institution accommodation, for the problem of tuberculosis lies largely among them." In discussing the scheme for the prevention and treatment of venereal diseases, Dr. Edwards states that although the number of patients attending the clinic has increased, there is no doubt that a considerable number of patients suffering from such diseases will not attend on account of its supposed publicity. This difficulty is certain to be met with in connection with such clinics in the smaller boroughs, and it militates against the practical application of the recent recommendation of the National Council for Combating Venereal Diseases that such clinics should be provided in all towns with a population exceeding 10,000. In the introduction to the report a plea is entered for the unification of public health effort under a Ministry of Health, and with what is said on this important subject we are in entire agreement.

